# Safety and efficacy of a proprietary thermogenic and cutting agent on measures of muscular strength and endurance, body composition, fat metabolism, and hormone levels

**DOI:** 10.1186/1550-2783-12-S1-P13

**Published:** 2015-09-21

**Authors:** Jacy Mullins, Jordan Outlaw, Stacie Urbina, Sara Hayward, Josh Holt, Bailey Burks, Alena Regelski, Eliza Fallice, Matt Stone, Colin Wilborn, Lem Taylor

**Affiliations:** 1 Department of Exercise & Sports Science, Human Performance Lab, University of Mary Hardin-Baylor, Belton, TX 76531, USA

## Purpose

The purpose of this study was to conduct a clinical trial on the commercially available thermogenic supplement (Iron Cuts^®^) and its effects on various markers of performance, metabolism, body composition, and hormone levels. The supplement evaluated in this study contains several ingredients (caffeine, green tea extract, fenugreek, etc.) that have been shown to promote positive adaptations of some of the dependent variables of interest.

## Methods

Twenty resistance-trained male subjects (21.10 ± 2.5 yrs, 177.4 ± 5.2cm, 87.2 ± 15.4kg, 14.8 ± 5.4 BF%) participated in a four-day per week split body resistance program. Participants were matched based on lean mass and randomly assigned to consume either a placebo (PL) or the dietary supplement Iron Cuts (IC). At baseline (PRE), subjects were assessed on body composition via DEXA, circumference measurements, 1 repetition maximum (1RM) and repetitions to failure on bench press and leg press. After concurrent training and supplementation for six weeks, all testing at baseline was repeated (POST). Subjects performed 3 consecutive supine resting energy expenditures and heart rate/blood pressure assessments before (0MIN), 30 minutes after (30MIN), and 60 minutes after (60MIN) ingesting an acute dose of the either PL or IC. Data were analyzed via ANOVA with repeated measures and statistical significance was accepted at p ≤ 0.05.

## Results

Significant time effects were observed for leg press (PRE: 925.0 ± 139.86; POST: 1089.1 ± 159.04lbs; p = 0.005) and bench press (PRE: 274.5 ± 73.8; POST: 290.9 ± 70.1lbs; p = 0.005) 1RM indicating the resistance training program was sufficient to induce changes in strength. A significant group by time interaction was observed for leg press 1RM (p = 0.05) indicating that strength gains were greater in the IC (Δ 1RM: 164.1lbs) versus PL (Δ 1RM: 88.9lbs) groups. Both groups improved lean muscle mass and percent body fat, but no significant effects were observed. A significant group by time interaction was observed with serum cortisol (p = 0.032), however, these changes observed were within normal clinical values. REE increased 6.8% and 12.34% in IC group and 5.7% and 5.2% in PL group at 30M and 60M, respectively. A significant time effect for REE (p = 0.005) and RQ (p = 0.017) was observed with no differences between groups. No significant changes were observed in circumference measurements of the biceps, thigh, chest, and waist.

## Conclusions

Based upon outcomes of this study, the supplement is apparently safe for both acute and chronic supplementation from a hemodynamic and blood analysis (CMP and CBC) perspective. Despite differential changes between groups in variables such as REE, lean muscle mass, and percent body fat, only lower body strength was shown to be improved via supplementation during resistance training. The reduction in serum cortisol in the IC group should be noted and evaluated with further research, but likely has little clinical significance.

